# Identifying relevant biomarkers of brain injury from structural MRI: Validation using automated approaches in children with unilateral cerebral palsy

**DOI:** 10.1371/journal.pone.0181605

**Published:** 2017-08-01

**Authors:** Alex M. Pagnozzi, Nicholas Dowson, James Doecke, Simona Fiori, Andrew P. Bradley, Roslyn N. Boyd, Stephen Rose

**Affiliations:** 1 CSIRO Health and Biosecurity, The Australian e-Health Research Centre, Brisbane, Australia; 2 The School of Information Technology and Electrical Engineering, The University of Queensland, Brisbane, Australia; 3 Stella Maris Scientific Institute, Pisa, Italy; 4 Queensland Cerebral Palsy and Rehabilitation Research Centre, School of Medicine and Science, Centre for Children’s Health Research, The University of Queensland, Brisbane, Australia; Chinese Academy of Sciences, CHINA

## Abstract

Previous studies have proposed that the early elucidation of brain injury from structural Magnetic Resonance Images (sMRI) is critical for the clinical assessment of children with cerebral palsy (CP). Although distinct aetiologies, including cortical maldevelopments, white and grey matter lesions and ventricular enlargement, have been categorised, these injuries are commonly only assessed in a qualitative fashion. As a result, sMRI remains relatively underexploited for clinical assessments, despite its widespread use. In this study, several automated and validated techniques to automatically quantify these three classes of injury were generated in a large cohort of children (n = 139) aged 5–17, including 95 children diagnosed with unilateral CP. Using a feature selection approach on a training data set (n = 97) to find severity of injury biomarkers predictive of clinical function (motor, cognitive, communicative and visual function), cortical shape and regional lesion burden were most often chosen associated with clinical function. Validating the best models on the unseen test data (n = 42), correlation values ranged between 0.545 and 0.795 (p<0.008), indicating significant associations with clinical function. The measured prevalence of injury, including ventricular enlargement (70%), white and grey matter lesions (55%) and cortical malformations (30%), were similar to the prevalence observed in other cohorts of children with unilateral CP. These findings support the early characterisation of injury from sMRI into previously defined aetiologies as part of standard clinical assessment. Furthermore, the strong and significant association between quantifications of injury observed on structural MRI and multiple clinical scores accord with empirically established structure-function relationships.

## Introduction

Cerebral palsy (CP) is an umbrella term that covers a range of injury occurring in the developing brain, pre- or perinatally [1]. Although impairments to motor function are the most characteristic symptom of CP [2], impairments to cognition, vision and communication have also been observed as a result of these injuries [3,4]. Magnetic resonance imaging (MRI) is strongly recommended to elucidate the presumed timing and aetiology of brain insults that cause CP [5] to help facilitate therapeutic selection [6], and is the current standard for assessing injury in clinical practice. Current characterisation of injuries related to CP are broadly grouped into three classes based on aetiological patterns [7]: brain maldevelopments (of which cortical malformations are the main type) occurring from disturbances in the first and second trimesters; periventricular white matter injury (leading to white matter lesions, and potentially the secondary ventricular enlargement due to primary tissue loss) occurring from disturbances in the early third trimester; and cortical/deep grey matter injury occurring from disturbances in the late third trimester (leading to grey matter lesions). The use of these characterisations are common, and many studies have investigated the prevalence of these timing-related aetiologies of injury within specific cohorts of children with CP [7–9]. However, these classifications have only been qualitatively assessed, disregarding the location and severity of lesions observed from the structural MRI (sMRI).

**Fig 1:**
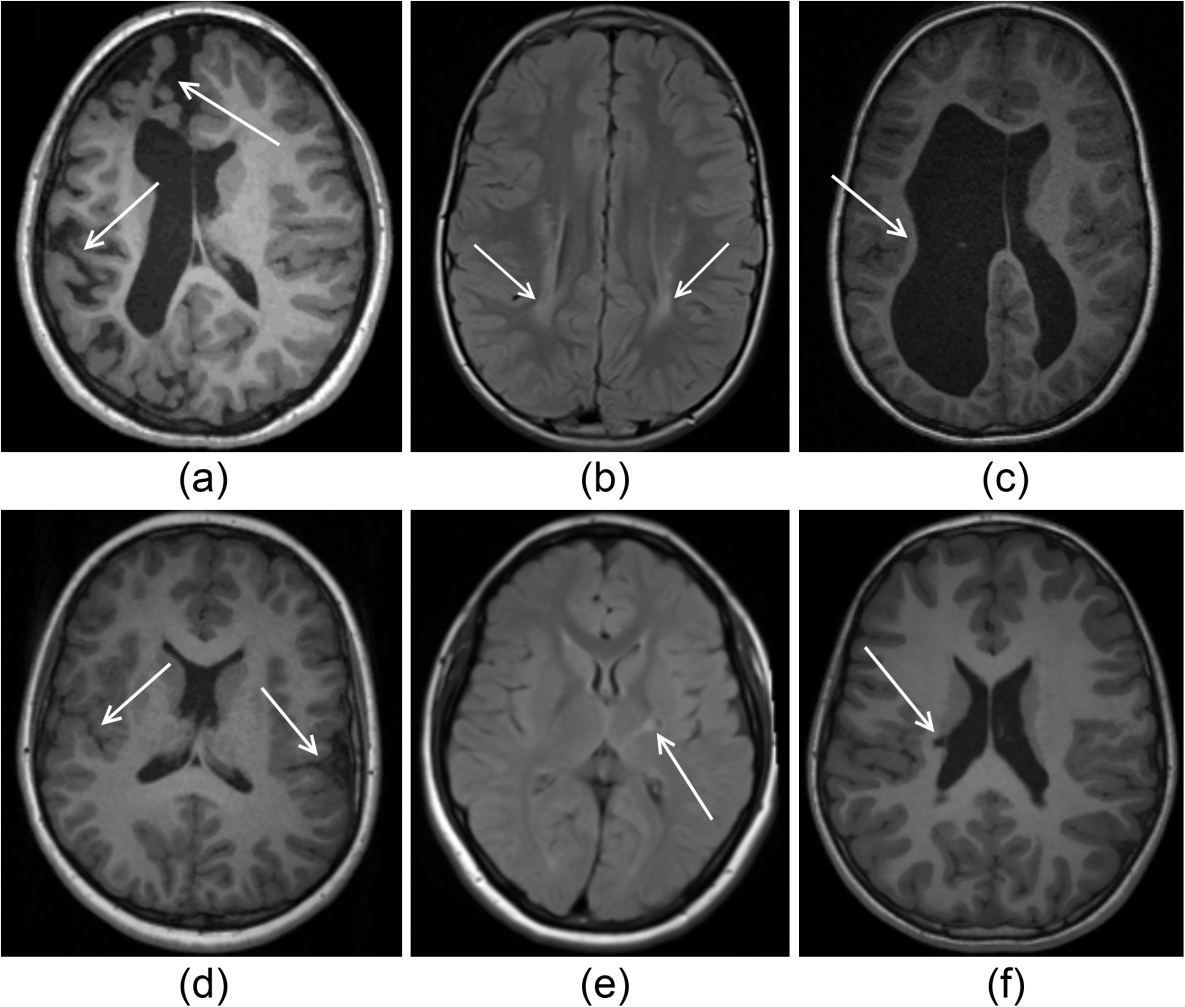
Illustrations of severe injury (top row) and subtle injury (bottom row) in cases of cortical malformations (first column), white/grey matter lesions (middle column) and ventricular enlargement (last column). (A) WM atrophy with corresponding ventricular enlargement is presented with abnormal sulcal depth predominantly in the frontal and occipital lobes. (B) WM lesions resulting from periventricular leukomalacia are shown as local regions of high intensity. (C) The consequences of a periventricular haemorrhagic infarction leading to a severe loss of WM and secondary enlargement of the lateral ventricles is shown, particularly on the left hemisphere. (D) Bilateral perisylvian polymicrogyria is shown, which are visible as excessive numbers of small gyri. (E) Gliosis in a small location in the posterior limb of the internal capsule is shown. (F) Periventricular cystic lesion in the right ventricular cella media is shown, leading to a small region of ventricular enlargement on the lateral side of the ventricle.

Quantitative measures of injury, accounting for injury severity and anatomical location, have the potential to better quantify the relationship between brain lesions and functional outcomes [10]. Furthermore, in future, such characterisations of injury may allow for valid and reliable predictions of patient impairment from MRIs acquired very early in life, which has implications for the selection of treatment strategies. However, this requires lesion volumes to be segmented in three dimensional images, which is too laborious to perform manually on large cohorts of data. Automated techniques are necessary to perform these segmentations in a repeatable and time-efficient manner. Such techniques need to be tailored to the specific challenges present in the sMRI of children with CP, including the potentially severe morphological alterations and the heterogeneous appearance of lesions, which are illustrated in Fig 1. However, no study has utilised the comprehensive quantifications of injury that automated approaches allow, nor have they examined the utility of using these quantifications to predict clinical function. As a result, it remains unclear whether quantitative measures of brain injury can usefully augment the current classifications of brain injury in the clinical setting to produce finer estimates of patient function. To this end, this study aims to quantitatively characterise the prevalence and extent of different manifestations of injury in a cohort of 139 children; including 95 children diagnosed with unilateral CP and 44 children with healthy development (CHD), using three previously developed automated approaches. These approaches identify the three main aetiologies of injury based on current classifications [11]; including a tailored segmentation strategy and cortical shape analysis pipeline to detect cortical malformations [12], a lesion-as-outlier segmentation strategy using T1- and T2-weighted MRIs for the detection of white and grey matter lesions [13], and a statistical shape model (SSM) of healthy ventricular shape to detect the secondary enlargement of ventricles [14]. The quantitative biomarkers derived from these approaches were used to characterise the prevalence of injury in this cohort of children with CP, and to construct regression models, in order to identify those biomarkers that are significantly associated with clinical scores of motor, cognitive, communicative and visual function. Such models can improve our understanding of the relationship between the extent and topography of brain lesions and clinical function in children with CP, and can provide valid and reliable estimates of patient function, which can help to tailor treatment strategies, potentially leading to improved gains in functional outcomes for children with CP.

## Methods

### Data

A total of 139 patients were included in this study: 95 patients with clinically diagnosed unilateral CP (50 male, 44 female, mean age 11.4, age range 5–17), and 44 CHD children (15 male, 29 female, mean age 10.7, age range 7–16) were included. Imaging data was made available from the Queensland Cerebral Palsy and Rehabilitation Research Centre (QCPRRC), as well as from the Stella Maris Institute, Pisa. Study participants included children who were recruited as part of ongoing studies of children with CP [15,16]. Diagnoses of CP were made based on clinical assessment by experienced clinicians in the field of CP. For both studies, ethical approval was granted and informed parental consent was obtained for all participants.

### Imaging acquisition

All 139 patients underwent T1 Magnetization Prepared Rapid Gradient Echo (MPRAGE) scanning from one of two different scanners, and one of three different scanning parameters, including a 3T Siemens’ scanner with scanning parameters (TR = 1900 ms, TE = 2.32 ms, flip angle = 9 degrees, slice thickness = 0.9 mm), and a 1.5T GE scanner with two different scanning parameters (TR = 12.36 ms, TE = 5.17 ms, flip angle = 13 degrees, slice thickness = 1 mm) and (TR = 124.29 ms, TE = 4.37 ms, flip angle = 10 degrees, slice thickness = 1 mm). The developed image processing algorithm is robust to imaging sequences, and has been validated previously on this dataset [17]. The influence on the Expectation Maximisation (EM)/ Markov Random Field (MRF) algorithm is minimal as it adaptively models the intensity distribution of each tissue class.

A subset of patients (*n* = 125) also underwent either T2 Turbo Inversion Recovery Magnitude (TIRM) (TR = 7000 ms, TE = 79 ms, flip angle = 120 degrees, slice thickness = 4 mm) or T2 Half-Fourier Acquisition Single-Shot Turbo Spin-Echo (HASTE) (TR = 1500 ms, TE = 81 ms, flip angle = 150 degrees, slice thickness = 4 mm), acquired using a 3T Siemens’ scanner.

### Clinical scores

Motor and cognitive impairments are the most common forms of disabilities in children with CP [3], although accompanying impairments to speech and vision have also been shown in up to 70% of patients [4]. Therefore, measures of patient motor, cognitive, visual and communicative function are all considered important in clinical assessment. As part of the ongoing studies on children with unilateral CP [15,16], six available clinical scores covering this potential range of functional impairments were Used in this study. The Assisting Hand Assessment (AHA) is a reliable measure of how well the assisting hand is used by the patient in bimanual tasks [18], and in this study is used to quantify the manual capabilities of children with CP. Measures of patient cognition, although difficult to quantify, is measured in this study using two parent reported questionnaires scoring their child’s behavioural and emotional function in daily life; the Behaviour Rating Inventory of Executive Function (BRIEF) [19] and Strengths and Difficulties Questionnaire (SDQ) [20]. To quantify visual acuity in this study, the Test of Visual Perception Skills (TVPS) measure [21] was utilised to assess patients’ ability to discriminate and memorise visual cues. Communicative ability was quantified in this study using the vocabulary (VOC) and word reasoning (WR) subtests of the Wechsler Preschool and Primary Scale of Intelligence (WPPSI-III) [22], which assess the patients’ ability to express and comprehend language, respectively.

### Demographic information

The demographics of the children with CP and the CHD children in this cohort are provided in Table 1 below. There were slightly less males compared with females in the CHD group (p = 0.042), and no difference in the ages between CHD and CP groups (p = 0.548).

**Table 1:** Demographic characteristics for the CHD and CP cohorts. For the CHD cohort, their clinical scores and injury severity score were not obtained.

Cohort	CHD cohort	CP cohort
Number of patients	44	95
Gender		
Male	15	50
Female	29	45
Age at scan (years)		
Mean ± standard deviation	11.73 ± 2.51	11.41. ± 3.08
Range (minimum—maximum)	7–16	5–17
Global brain injury severity score [23]		
Mean ± standard deviation	0.00 ± 0.00	9.20 ± 4.88
Range (minimum—maximum)	0–0	1–21
Assisted Hand Assessment (AHA) Score		
Mean ± standard deviation	NA	64.55 ± 2.08
Range (minimum—maximum)	NA	8–98.8

CHD, children with healthy development; GM, grey matter; NA, not available; WM, white matter.

### Image pre-processing

Several image pre-processing steps were performed on the T1-MPRAGE, T2-TIRM or T2-HASTE images prior to the extraction of biomarkers of injury. Firstly, image alignment to the Colin 27 Average Brain Atlas [24] using an affine block matching registration algorithm [25]. Image bias was corrected for using the N4 algorithm [26]. Intensity normalisation and image de-noising, using anisotropic diffusion [27] with modified curvature diffusion equation [28], was performed with the Insight Toolkit (ITK) in order to minimise the effect of Rician-distributed noise in the MR image, while attempting to preserve high resolution features within the image. Skull stripping of the T1-MPRAGE, T2-TIRM and T2-HASTE images was performed using an in-house algorithm developed in MATLAB (Mathworks, Natick, MA). In this approach, intradural CSF was identified using thresholding and morphological operations, following which brain tissues were isolated. By identifying CSF internal to the skull boundary, this approach is capable of accurately segmenting the brain in cases of large lesions, which may be present in children with CP. Tissue probability maps (TPMs) from the Colin 27 Atlas were registered to the T1-weighted MRIs of each patient using the fast free-form deformation registration algorithm [29] to assist the downstream lesion segmentation.

### Image biomarkers

**Fig 2:**
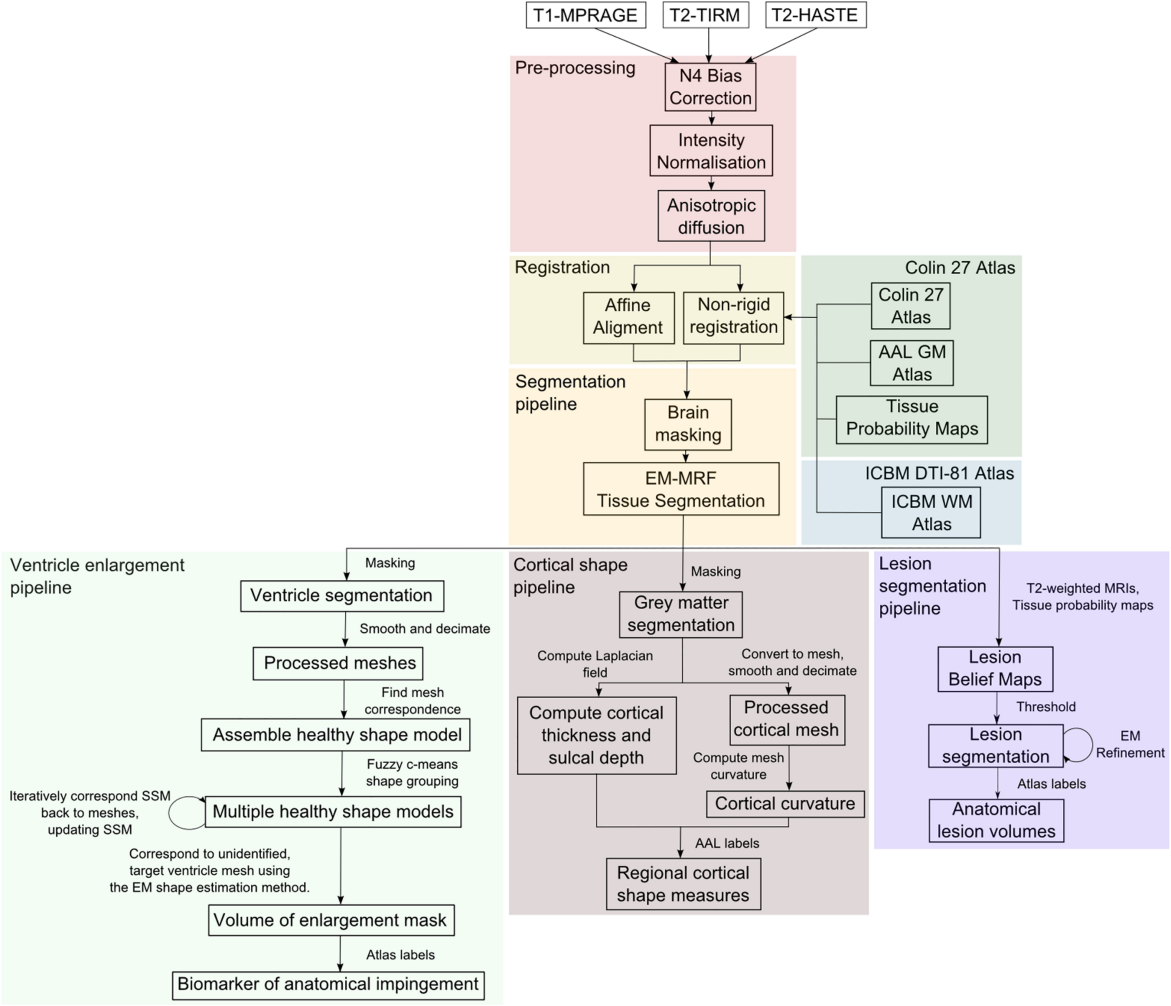
Illustration of the overall processing pipeline. The pre-processing steps are shown in red, registration steps in yellow, the utilised atlases in green and blue, and the brain masking and tissue segmentation approaches in orange which facilitate the detection of ventricular enlargement (shown in light green), cortical shape measures (in brown) and lesion burden (in purple).

Image-processing techniques were used to identify and quantify three types of injury observed in the MRIs of children with CP [11]. These methods and their performance are detailed in the following sections. An overview of the pipeline of these automated methods are illustrated in Fig 2.

### Identifying cortical malformations

**Fig 3:**
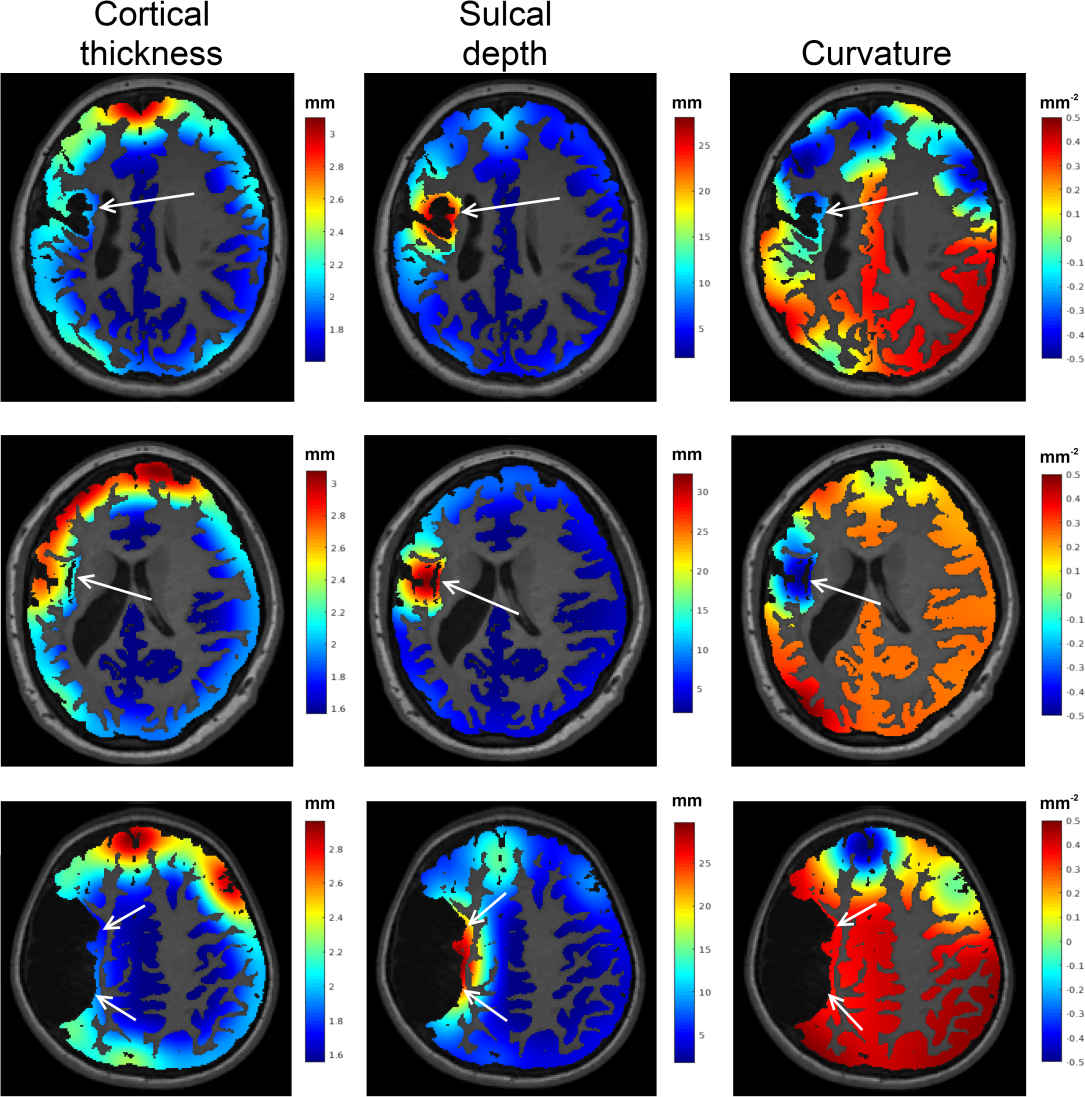
An illustration of three cortical abnormalities. The first column shows the measured cortical thickness (in mm), the second columns shows sulcal depth (in mm) and the third column shows the curvature (in mm^-2^) of these cortices. Regions of injury were observed to have higher sulcal depths, and reduced cortical thickness and curvature.

Primary (i.e. brain maldevelopments) or secondary (i.e. due to hypoxic insult) cortical abnormalities, which appear as a heterogeneous range of altered cortical shapes, were identified in this study using a brain tissue segmentation algorithm that accommodates severe injury [17], and three shape measures computed from the cortical grey matter segmentation (cortical thickness, curvature, sulcal depth) in order to quantify shape abnormalities [12] in each cortical region defined by the Automated Anatomical Labelling (AAL) atlas. An illustration of these cortical measures in three patients with observed cortical alterations are shown in Fig 3. Note for these figures, the cortical measures, which were obtained only on the cortical surface, were smoothed across the entire cortical grey matter segmentation. Biomarkers obtained from this approach are an absolute z-score from healthy cortical shape measures measured from the corresponding cortical region among the 44 CHD children. Unlike an absolute measure of cortical shape (such as cortical thickness in millimetres), a z-score relative to healthy cortical shape aligns with the subsequent volumes of lesion burden and ventricular enlargement volume, where larger values represent greater injury severity. This also facilitates the interpretation of the regression model coefficients.

### Identifying white and grey matter lesions

**Fig 4:**
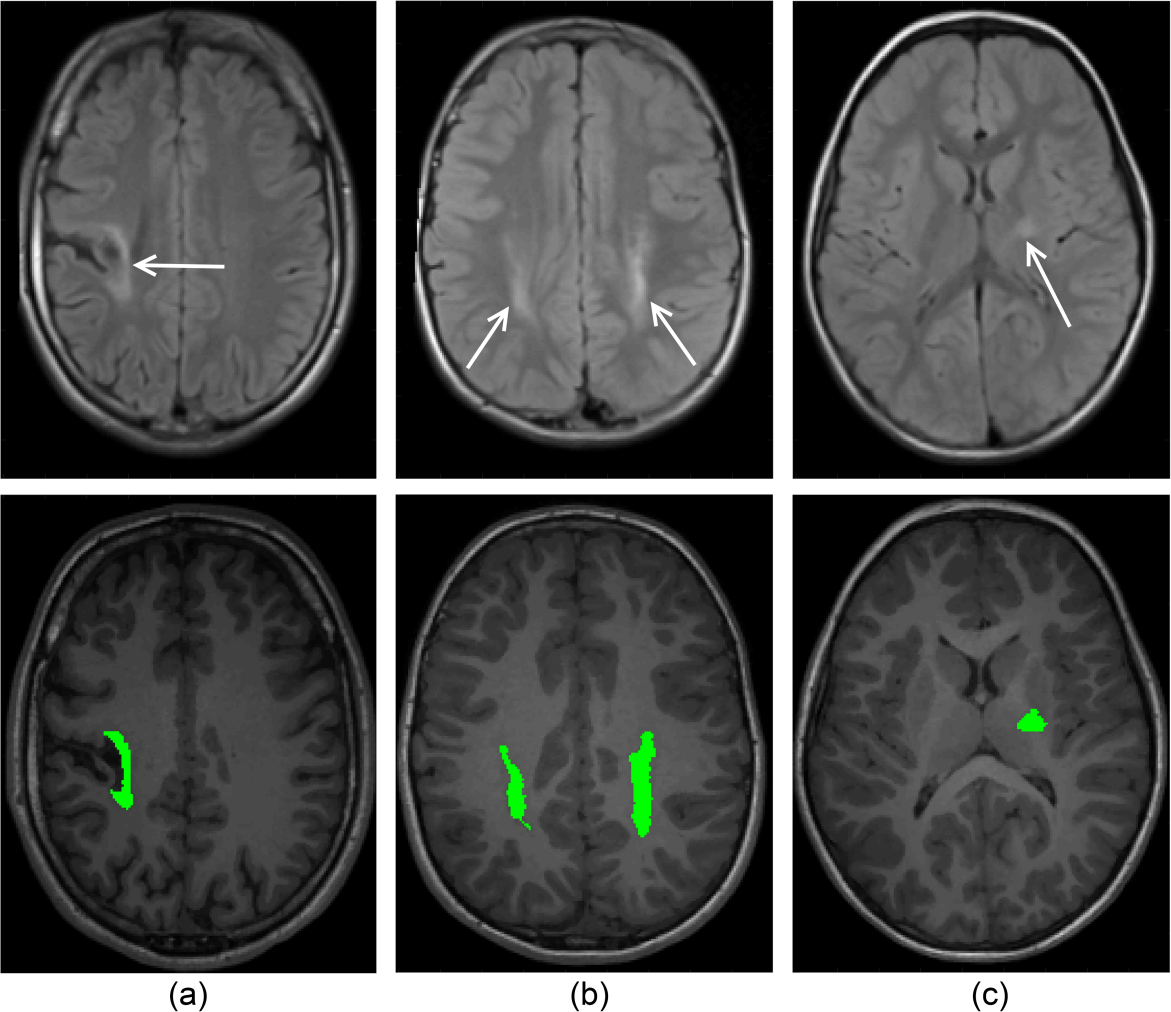
Illustration of lesion segmentation. (A) GM lesion segmentation, (B), WM lesion segmentation and (C) internal capsule lesion segmentation.

White and grey matter lesions were identified using a lesion segmentation algorithm tailored to CP data, and with multiple lesion classes to identify both types of lesions [13]. Biomarkers from this approach are a lesion volume (in mL) in the different brain regions, identified using the AAL grey matter atlas and the ICBM DTI-81 white matter parcellation atlas (International Consortium for Brain Mapping, CA) respectively. This approach achieved a sensitivity of 94% and a specificity of 93% for both white and grey matter lesions [13]. Although it was observed that the specificity of WM lesion segmentations alone were comparatively lower due to more frequent variations in WM intensity, while the sensitivity of GM lesion segmentations alone were comparatively lower due to reduced contrast observed for GM lesions. Illustrations of white and grey matter lesion segmentations are shown in Fig 4.

### Identifying ventricular enlargement

**Fig 5:**
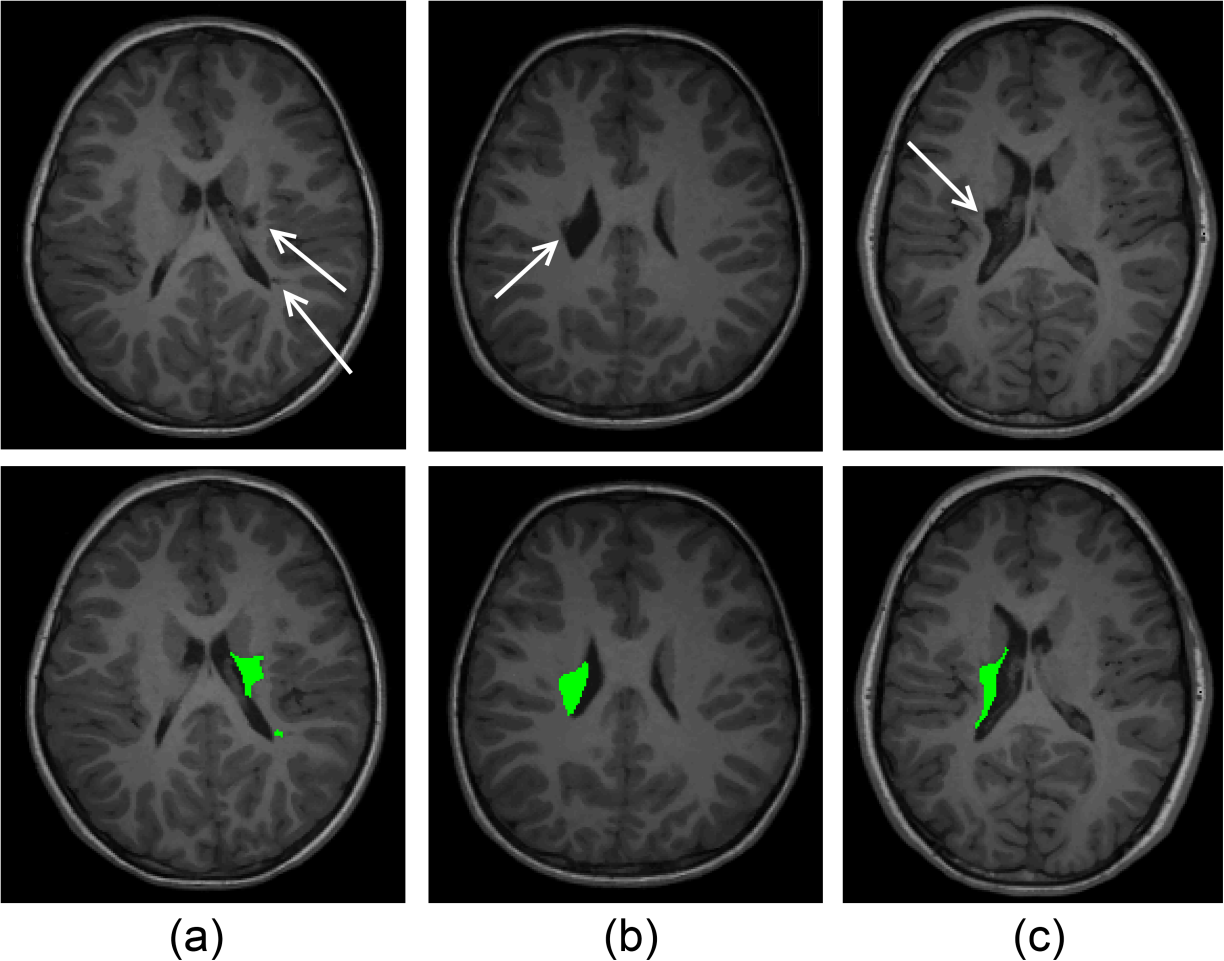
Illustration of three cases of localised enlarged ventricles due to injury, and the segmentation of the enlarged volumes from the nearest healthy ventricle shape.

Ventricular enlargement was identified using a SSM of healthy lateral ventricles to extract volumes of enlargement, and compute their impingement on nearby deep grey matter anatomies [14]. Biomarkers from this approach include a volume of ventricular enlargement (in mL) in the deep grey matter anatomies, as determined by the AAL grey matter atlas. Note that this is not the entire lateral ventricular volume, solely the volume of the region that is thought to be enlarged, compared to typically developing children. Three cases illustrating the segmented regions are shown in Fig 5.

### Statistical methodology

The Least Absolute Shrinkage and Selection Operator (LASSO) method [30] was used to define optimal sets of relevant biomarkers of brain injury associated with patient outcomes. Data was partitioned, with models trained on 75% of the data and their performance validated on the independent 25% test set. In the LASSO approach, predictor variables that are not strongly associated with the clinical outcomes are removed from the model. The LASSO was run with default lambda and alpha parameters that define the weight, and type of the regularisation penalty respectively. Patient age and gender were included in all regression models, as it has been previously shown that there are age [31] and gender [32] impacts on both motor and cognitive outcomes. To account for the variance in different scanner sequences used in this study, scanner sequence was also included as a covariate in each model.

The regression coefficients of each model, which encode the relative weights of individual biomarkers impacting clinical outcome, were enforced to be negative. This was done in order to only extract correlations where observed injury led to reductions in patient outcome. In total, six models were constructed, one for each of the six clinical scores (AHA, BRIEF, SDQ, TVPS, WR and VOC). Multiple comparisons of the models were corrected for using a Bonferroni correction. An analysis of variance analysis (ANOVA) was performed to compare the complete models from all biomarkers combined, and the biomarkers of each type of injury individually, to ascertain if it is beneficial to look at all kinds of injury observed in the MRIs concurrently. All statistical analysis were conducted using the R statistical software Version 3.2 [33].

## Results

### Characterisation of injury in the cohort

**Fig 6:**
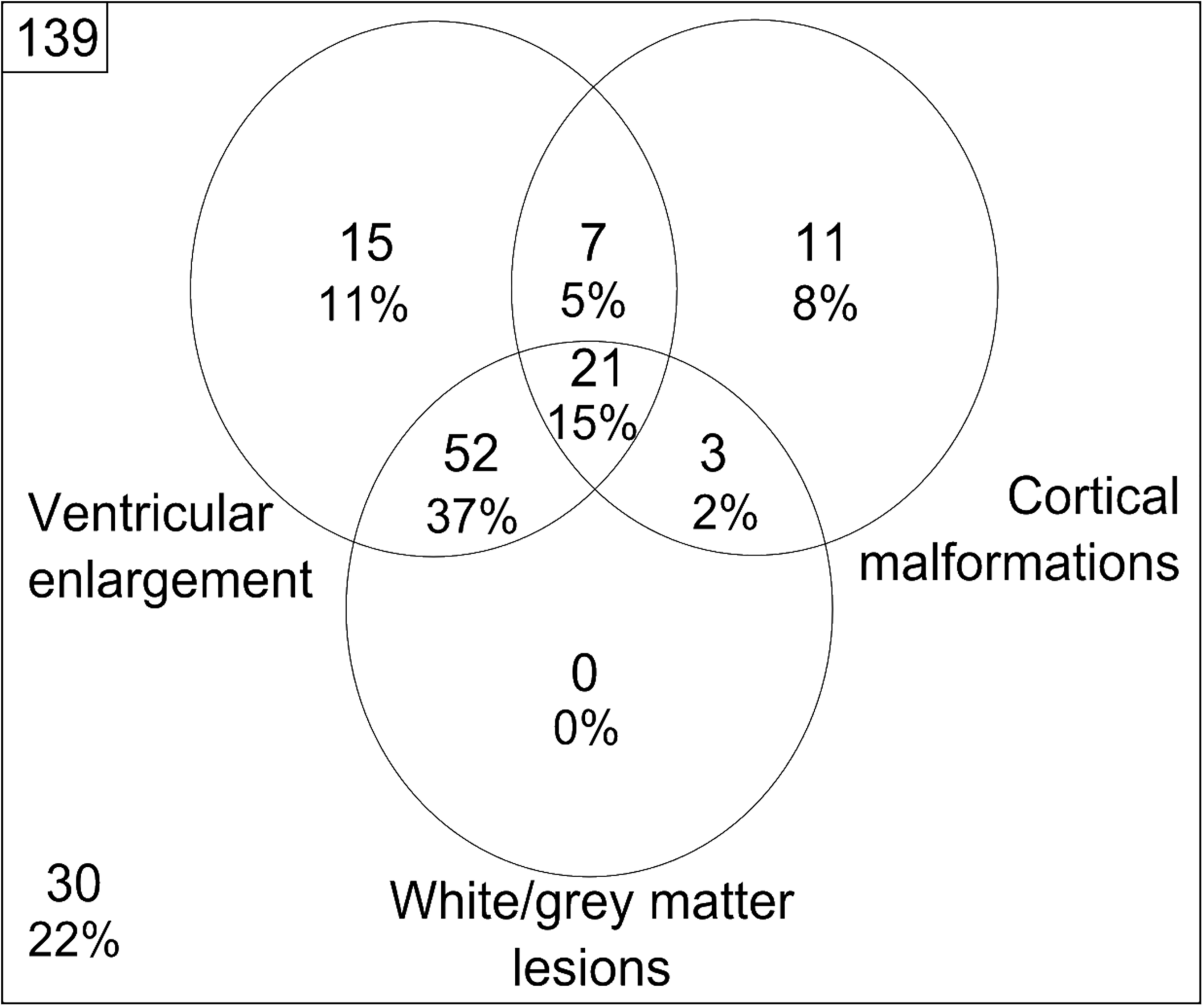
A Venn diagram characterising the observed prevalence of ventricular enlargement, cortical malformations and white/grey matter lesions observed in our cohort using the described automated techniques.

The prevalence of injury, as determined using the automated analyses, in the cohort of children with CP is illustrated in Fig 6. Prevalence is shown as a Venn diagram, to illustrate that each individual patients may have a combination of the three classes of injury. Binary characterisation of injury was classified as the presence of any enlarged ventricles or white/grey matter lesions (greater than 0 mL anywhere in the brain), or a regional cortical shape with a computed z-score > 2.5 compared to corresponding healthy cortical shape (for any cortical shape and region). Of the 139 children in our cohort, 109 were classified as having some form of injury, with 15% of the cohort having all three types of injury identified. Ventricular enlargement was the most common form of injury (observed in 68% of children), followed by white/grey matter lesions (55%) and cortical abnormalities (30%). As expected from the known aetiology of injury, ventricular enlargement was observed in over 60% of patients with white/grey matter lesions. False positive characterisations of ventricular enlargement, caused by slight alignment errors of the ventricular meshes, explains why 14 children with typical brain development were incorrectly identified as possessing some form of brain injury.

### Observed structure-function relationships

The image biomarkers retained from LASSO, and their respective regression coefficients for each of the six models, are given in Table 2 below. In these models, the cortical shape biomarkers (labelled ‘Cortical thickness’, ‘Curvature’ or ‘Sulcal depth’) represent the reduction in clinical outcome for a 1 unit increase in z-score from healthy shape in that cortical region, while the lesion biomarkers (labelled ‘Lesion’) and ventricular enlargement biomarkers (labelled ‘Ventricle enlargement’) represent the decrease in clinical score from an increase in 1mL of lesion, or enlarged ventricle respectively, in that anatomy. In this table, we note that the p-values of each biomarker are uncorrected, and simply reflect the strength of that feature within the chosen model. However, the adjusted R-squared of the models, which measure strength of the correlation between the outcome and the model predictions, were compared against a Bonferroni corrected alpha value (0.05/6 tests, 0.008).

**Table 2:** The retained anatomical regions, and corresponding regression coefficients (including standard errors) of the six regression models modelled on the 75% training set. For each model, the multiple R-squared is provided. Model features that are significant (*p* < 0.05) are bolded.

**AHA**		
*Independent variable*	*Regression coefficient*	*Standard error*
**(Intercept)**	116.80***	4.741
Supplementary motor area—Curvature	-7.462	2.828
**Primary somatoensory cortex—Curvature**	-15.157**	3.810
**Cingulate—Cortical thickness**	-14.063**	3.259
**Lingual gyrus—Sulcal depth**	-13.062*	4.306
**Middle temporal gyrus—Sulcal depth**	-14.887**	2.566
**Lenticular nucleus—Lesion**	-0.051*	0.017
**External capsule—Lesion**	-0.012**	0.003
Cerebral peduncle—Lesion	-0.044	0.020
Age	0.302	0.599
Gender (Reference: Male)	1.733	3.547
MR Sequence (Reference: QCPRRC)	-4.850	4.820
Adjusted R-squared	0.728***	
**BRIEF**		
*Independent variable*	*Regression coefficient*	*Standard error*
**(Intercept)**	202.195***	19.884
Primary somatosensory cortex—Sulcal depth	-11.116	6.205
Insula—Curvature	-15.949	9.506
Occipital gyrus—Curvature	15.342	9.1775
**Inferior temporal gyrus—Curvature**	-21.469*	7.685
Premotor cortex—Cortical thickness	-12.619	9.222
Middle temporal gyrus—Cortical thickness	-16.595	9.875
Middle frontal gyrus—Lesion	-0.049	0.033
Caudate nucleus—Lesion	-0.053	0.038
Age	1.267	1.662
Gender (Reference: Male)	18.522	8.958
MR Sequence (Reference: QCPRRC)	2.499	12.254
Adjusted R-squared	0.310*	
**SDQ**		
*Independent variable*	*Regression coefficient*	*Standard error*
**(Intercept)**	81.750***	5.252
**Primary motor cortex—Cortical thickness**	-2.392*	1.099
Primary motor cortex—Sulcal depth	-1.286	0.808
**Insula—Curvature**	-4.839**	1.239
Cingulate cortex—Cortical thickness	-2.656	1.090
**Cingulate cortex—Curvature**	-1.631*	0.527
Fusiform gyrus—Curvature	-1.057	0.774
Lingual gyrus—Curvature	-2.268	1.161
Lingual gyrus—Sulcal depth	-1.238	5.313
**Inferior temporal gyrus—Sulcal depth**	-4.963***	0.973
Premotor cortex—Cortical thickness	-3.196	1.368
**Middle temporal gyrus—Curvature**	-4.221*	1.207
Superior occipital gyrus—Lesion	-0.090	0.059
Superior temporal gyrus—Lesion	-0.005	0.003
**Corona radiata—Lesion**	-0.001**	<0.001
Age	0.222	0.242
Gender (Reference: Male)	1.835	1.615
MR Sequence (Reference: QCPRRC)	0.424	1.554
Adjusted R-squared	0.707***	
**TVPS**		
*Independent variable*	*Regression coefficient*	*Standard error*
**(Intercept)**	135.004***	10.693
Primary motor cortex—Cortical thickness	-7.782	5.024
Rolandic operculum—Cortical thickness	-2.814	3.447
Supplementary motor area—Curvature	-4.382	3.599
Primary sensory cortex—Sulcal depth	-2.557	3.536
Insula—Sulcal depth	-2.248	0.981
Cingulate cortex—Curvature	-2.234	1.915
Fusiform gyrus—Cortical thickness	-18.213	15.334
Superior temporal gyrus—Cortical thickness	-3.800	6.289
Primary visual cortex—Cortical thickness	-7.382	5.339
Lingual gyrus—Curvature	-1.570	4.327
Inferior temporal gyrus—Sulcal depth	-4.191	3.650
Inferior frontal gyrus—Cortical thickness	-4.720	4.499
Middle temporal gyrus—Cortical thickness	-2.192	5.176
Middle frontal gyrus—Lesion	-0.019	0.015
Hippocampus—Lesion	-0.264	0.395
Superior occipital gyrus—Lesion	-0.295	0.238
Supramarginal—Lesion	-0.019	0.019
Caudate nucleus—Lesion	-0.008	0.019
Posterior thalamic radiations—Lesion	-0.002	0.004
Age	0.487	1.102
Gender (Reference: Male)	3.421	5.340
MR Sequence (Reference: QCPRRC)	0.322	6.475
Adjusted R-squared	0.577***	
**WR**		
*Independent variable*	*Regression coefficient*	*Standard error*
**(Intercept)**	96.247***	10.067
Cingulate cortex—Cortical thickness	-1.295	3.420
Cingulate cortex—Curvature	-0.734	1.395
Fusiform gyrus—Curvature	-0.930	2.292
Angular gyrus—Cortical thickness	-1.054	4.395
Primary visual cortex—Cortical thickness	-6.516	3.584
Primary visual cortex—Sulcal depth	-5.695	8.091
Cuneus—Curvature	-1.557	2.771
Lingual gyrus—Sulcal depth	-8.600	17.606
**Occipital gyrus—Curvature**	-11.531*	3.856
Inferior temporal gyrus—Curvature	-3.649	2.288
Inferior temporal gyrus—Sulcal depth	-2.910	2.340
Middle frontal gyrus—Cortical thickness	-3.372	2.852
Gyrus rectus—Curvature	-1.219	2.088
Precentral gyrus—Lesion	-0.003	0.005
External capsule—Lesion	-0.002	0.003
Cerebral peduncle—Lesion	-0.003	0.016
**Age**	-2.272**	0.532
Gender (Reference: Male)	-3.423	2.39
**MR Sequence (Reference: QCPRRC)**	-6.935*	3.115
Adjusted R-squared	0.385*	
**VOC**		
*Independent variable*	*Regression coefficient*	*Standard error*
(Intercept)	8.980	22.027
Supplementary motor area—Curvature	-0.810	2.795
Primary somatosensory cortex—Cortical thickness	-7.609	3.801
Insula—Sulcal depth	-0.364	0.871
Cingulate cortex—Curvature	-0.674	1.341
Angular gyrus—Curvature	-0.713	4.449
Angular gyrus—Sulcal depth	-0.919	1.429
Primary visual cortex—Cortical thickness	-2.845	4.301
Lingual gyrus—Curvature	-3.136	3.246
Inferior temporal gyrus—Sulcal depth	-0.979	3.168
Posterior parietal gyrus—Sulcal depth	-5.267	2.609
Gyrus rectus—Cortical thickness	-5.610	3.997
Middle temporal gyrus—Cortical thickness	-1.371	3.507
Middle frontal gyrus—Lesion	-0.014	0.019
Inferior frontal gyrus—Lesion	-0.006	0.009
Cingulate cortex—Lesion	-0.011	0.062
Hippocampus—Lesion	-0.325	0.183
Middle occipital gyrus—Lesion	-0.017	0.016
Caudate nucleus—Lesion	-0.020	0.014
Posterior thalamic radiations—Lesion	-0.002	0.004
Cingulum—Lesion	-0.034	0.249
**Age**	4.090***	0.805
Gender (Reference: Male)	0.608	3.013
MR Sequence (Reference: QCPRRC)	-2.695	3.807
Adjusted R-squared	0.575***	

Correlations in bold have a statistical significance of p < 0.008. Asterisked correlations were found to be statistically significant after multiple comparisons

* *p* < 0.008

** *p* < 0.0016

*** *p* < 0.00016.

AHA, Assisting Hand Assessment; BRIEF, Behaviour Rating Inventory of Executive Function; GM, grey matter; PLIC, posterior limb of the internal capsule; SDQ, Strengths and Difficulties Questionnaire; TVPS, Test of Visual Perception Skills; VOC, vocabulary; WM, white matter; WR, Word reasoning

### Analysis of predictive biomarkers

The performance of the models on the unseen test set, shown in Table 3, demonstrates how well these observed relationships could predict functional outcomes in children with observed brain injury. Five of the six data-driven models were statistically significant (*p* < 0.008) in the 25% test set. Although the correlations for the manually chosen biomarkers were generally smaller than the corresponding data-driven models, four of these six models were statistically significant (*p* < 0.008).

**Table 3:** Pearson’s R correlation between the predicted outcomes in the test set using the trained regression models and the clinical scores of the test set, for both the data-driven and manually chosen models.

	*Pearson’s R correlation*	*Relative mean error (%)*	*95% Confidence Interval*
AHA	0.706**	0.145	(0.488, 0.841)
BRIEF	0.482	-0.303	(0.124, 0.729)
SDQ	0.795**	-0.419	(0.456, 0.932)
TVPS	0.617*	0.099	(0.252, 0.827)
WR	0.545*	-0.686	(0.161, 0.786)
VOC	0.682**	0.343	(0.354, 0.860)

Correlations in bold have a statistical significance of p < 0.008. Asterisked correlations were found to be statistically significant after multiple comparisons:

* *p* < 0.008

** *p* < 0.0016.

AHA, Assisting Hand Assessment; BRIEF, Behaviour Rating Inventory of Executive Function; SDQ, Strengths and Difficulties Questionnaire; TVPS, Test of Visual Perception Skills; VOC, vocabulary; WR, Word reasoning

### Assessment of independence of biomarker-type

To assess the independence of the different categories of biomarkers, an ANOVA was performed to compare the complete models obtained from the data-driven variable selection, and models constructed using only the biomarkers from this model of a specific injury class (i.e. cortical biomarkers and lesion biomarkers separately). The summary of these analyses is provided in Supplementary Table 1. Model comparisons to the ventricle enlargement biomarkers alone were omitted as no ventricular enlargement biomarkers were retained by LASSO in any model. All models containing only cortical shape or lesion burden biomarkers were found to be significantly different from the complete regression models (*p* < 0.05), except for the cortical biomarker only model for TVPS (*p* = 0.109). This suggests that the two different sets of biomarkers explain different portions of variance in the clinical score of children with CP.

## Discussion

The prevalence of brain injury observed in the present study using the automated approaches was found to be comparable to studies on other cohorts of children with CP [8,9,11]. Leveraging the quantifications that these automated methods allow, models of brain injury to patient function were generated. Five of the six models were observed to obtain significant correlations in this test set, demonstrating the clinical utility of these predictive models in estimating patient function in order to provide early and effective therapeutic interventions. Only the Brief model did not achieve significance, which may be due to reduced executive outcomes manifesting primarily as the loss of white matter connectivity, which would require diffusion MRI and tractography to identify [34]. On its own sMRI does not assess neural connectivity and the inclusion of diffusion data may result in greater association of these outcomes. However, unlike sMRI, there is not yet a routine and widely accepted method for diffusion MRI acquisition and processing of data [35]. These findings takes steps towards establishing standardised biomarkers used in the assessment of children with CP, with implications on estimating patient function, allowing effective interventions to be performed early in life, and to characterise structural brain changes as a result of therapy, improving the understanding of neuroplastic mechanisms.

Although the model features were retained from a data-driven process, the significant regions (p<0.05) conform to known structure-function relationships of the brain. For instance, the supplementary motor area has known associations with intended voluntary action [36], the primary somatosensory cortex has been associated with the mental rehearsal of motor acts [37], the anterior cingulate has known motor regions regulating the interactions between cognitive and motor control [38], supporting the observed importance of these regions in the AHA model (Table 2). Additional significant regions in the AHA model include the lenticular nucleus, which is known to control a variety of movements [39], and is connected to the external capsule [40], and the cerebral peduncle, which contains WM fibres of the corticospinal tract and has previously been shown to be predictive of motor deficits [41]. For the Brief and SDQ models of cognitive function, several of the retained regions (inferior temporal gyrus, primary motor cortex, insular cortex, cingulate cortex, premotor cortex and corona radiata) have known associations with cognition and executive functioning. For instance, the inferior temporal gyrus, which was significantly retained in both models, has a known role in verbal fluency and cognition, and is commonly affected in AD [42]. The observation in the Brief model that girls had higher reported executive functioning than boys may be due to females maturing earlier than males [43], or arising from the currently unexplained phenomenon that males with CP tend to exhibit more severe impairment [44]. Other significant features retained in the SDQ model include the primary motor and premotor cortices, which have both been shown to be responsible for motor learning and cognitive actions [45,46], the cingulate cortex that has been associated with the regulation of varied mental and emotional activity [47], and the corona radiata which has been associated with mental calculation and information processing [48]. The insular cortex, which was retained in both models for cognition (SDQ) and vision (TVPS), has known associations with multiple sensory areas, influencing awareness of somatosensation and goal directed cognition [49], and the processing of visual information [50]. We hypothesise that the presence of the occipital gyrus in the word reasoning model may arise from the known roles in the auditory and spatial information and stimuli [51], including sound localisation, which are required to perform well in the WPPSI-III subtests for communication. Age was retained in both models for vocabulary (VOC) and word reasoning (WR), potentially reflecting the known improvements in articulation [52] and phonological skills [53] occurring during childhood development.

Ventricular enlargement biomarkers were observed to be removed by LASSO’s variable selection for all models of clinical function. This may due to ventricular enlargement being a secondary injury caused by primary periventricular white matter tissue loss [54,55]. Consistent with this aetiology, most children with ventricular enlargement in this cohort had white or grey matter tissue lesions. As a result, both ventricular enlargement and lesion biomarkers explain similar portions of variance in these children’s functional outcomes. Since the ventricular enlargement biomarkers were removed by LASSO, this indicates that they explain relatively less variance in the functional outcomes than the lesion biomarkers. However, it was observed that both cortical morphology and lesion burden variables were retained by LASSO for all six models. The independence of these biomarkers is further demonstrated in the ANOVA supplementary table, identifying that the two sets of factors explain independent portions of variance in five of the six models. These findings highlight the benefit of quantifying both biomarkers for the assessment of MRIs.

Although the developed automated approaches were designed to be robust to the presence of severe brain injury, there are a number of technical limitations with these methods. Firstly, despite most of these approaches requiring only an affine alignment of atlases, which is mostly driven by the alignment of the skull, there are registration errors introduced in the deformable registration of the tissue probability maps used in the lesion segmentation pipeline. The brain masking segmentation step occasionally incorporates dura into the brain mask and miss subtle cortical sulci. In these cases, manual editing of the segmentations is required, as instances of either error will affect the cortical shape measures computed in the cortical analysis pipeline. The presence of slight correspondence errors between the ventricular shape models led to a number of false positive classifications of ventricular enlargement. Due to the risk of over-fitting on the limited available training data, no interaction terms were included for account for potential feature covariance, nor was WM connectivity information incorporated in the current study. The inclusion of diffusion data the model will be important in the future, because structural MRI alone cannot account for the variable influence of neuroplastic mechanisms that lead to altered structure-function relationships to compensate for the presence of injury [56,57]. These altered relationships confound the relationships between injury and impairment that the regression models attempt to elucidate, introducing unexplained variance in the clinical scores and potentially reducing the multiple R-squared of these models. Performing WM tract labelling, which has previously been achieved using label fusion [58], regional diffusion measures such as Fractional Anisotropy (FA) and Mean Diffusivity (MD) can be computed and incorporated into the regression models presented here. Diffusion measures such as FA and MD in key regions, such as the corpus callosum, have been associated with neurocognitive performance [59,60]. In the future, similar approaches can leverage longitudinal information when it becomes available, such as MRI taken early in life (around 2 years of age) and developmental trajectories through early childhood, in order to provide estimates of long-term function, which have been performed in the context of Alzheimer’s disease progression [61].

The main strength of this study is that the developed injury segmentation approaches only requiring well established T1-weighted sequences. More sophisticated diffusion and functional sequences are not as widely available, and have longer scanning times which complicate the imaging of young children. The development of automated techniques that only require these rapid and established sequences help facilitate potential translation to clinical practice. Secondly, this study encapsulates the full range of primary and secondary injuries characterised previously [7], as well as patient age and gender, allowing for comprehensive characterisation of brain injury from sMRI. In future, image analysis in the CP setting needs to move towards more automated quantification of injury, as it allows quantitative relationships between brain injury and outcome to be elucidated for children with both unilateral and bilateral CP. These associations have the potential to build upon the understanding of the brains structure-function relationships, which is an important prerequisite for understanding reorganisation and plasticity. Furthermore, there is potential in using these associations to produce estimates of patient function, which have important clinical implications for dictating the type and intensity of intervention that may be required, early in life in order to optimise functional outcomes.

## Conclusion

In this study, several validated, automated approaches for identifying three different aetiologies of brain injury were applied to a cohort of children diagnosed with unilateral CP. A similar prevalence of cortical malformations, white and grey matter lesions and secondary ventricular enlargement were observed to previous studies on populations of children with CP. Significant correlations were observed between the biomarkers of injury and multiple scores of patient motor, cognitive, visual and communicative function. These structure-function relationships generalised to unseen data, with correlations between 0.482 and 0.795, and retained predictor variables that conformed to known roles of brain structures. There was significant independence between cortical morphology and tissue lesions in explaining functional outcomes, highlighting the benefit of quantifying both of these types of injury for clinical assessment. Furthermore, the strong and significant association between quantifications of injury obtained from on structural MRI and multiple clinical scores agree with empirically established structure-function relationships. These findings support the early elucidation and characterisation of brain injury from sMRI in the clinical assessment of children with CP. In future, the methods detailed in this paper could facilitate the longitudinal assessment of patients during atypical development and rehabilitation, and will assist in quantifying structural changes due to therapeutic effects. As a result, the selection of optimal treatment strategies for individual cases can be performed, to ultimately improve outcomes for children with CP.

## Supporting information

S1 Table Analysis of independence between cortical morphology and lesion burden. ANOVA model comparisons between the complete regression models, and the models constructed with the cortical shape and lesion burden biomarkers only.
(PDF)
