# Supplementary material for: Identifying relevant biomarkers of brain injury from structural MRI: Validation using automated approaches in children with unilateral cerebral palsy
Source: PLoS One. 2017 Aug 1;12(8):e0181605. doi: 10.1371/journal.pone.0181605 (PMC5538741; doi:10.1371/journal.pone.0181605)
Supplement: S1 Table — ANOVA model comparisons between the complete regression models, and the models constructed with the cortical shape and lesion burden biomarkers only. (PDF) [file pone.0181605.s001.pdf]

Supplementary material

Analysis of independence between cortical morphology and lesion burden

S1 Table. ANOVA model comparisons between the complete regression models, and the models constructed with the cortical shape and lesion burden biomarkers only.

			**AHA**		
	Residual Sum of Squares	*Degrees of Freedom*	*Mean Square*	*F*	*Significance*
Complete model	7161.1	-	-	-	-
Cortical biomarkers only	12478.6	-3	-5317.5	12.623	<0.001***
Lesion biomarkers only	20031.5	-5	-12870	18.332	<0.001***
			**BRIEF**		
	Residual Sum of Squares	*Degrees of Freedom*	*Mean Square*	*F*	*Significance*
Complete model	26360	-	-	-	-
Cortical biomarkers only	32537	-3	-6177.1	3.125	0.036*
Lesion biomarkers only	44323	-7	-17963	3.894	0.002**
			**SDQ**		
	Residual Sum of Squares	*Degrees of Freedom*	*Mean Square*	*F*	*Significance*
Complete model	122.92	-	-	-	-
Cortical biomarkers only	276.38	-4	-153.46	6.554	0.001**
Lesion biomarkers only	627.53	-12	-504.61	7.184	<0.001***
			**TVPS**		
	Residual Sum of Squares	*Degrees of Freedom*	*Mean Square*	*F*	*Significance*
Complete model	3704.2	-	-	-	-
Cortical biomarkers only	5131.4	-6	-1427.2	1.927	0.109
Lesion biomarkers only	7877.5	-13	-4173.3	2.600	0.015*
			**WR**		
	Residual Sum of Squares	*Degrees of Freedom*	*Mean Square*	*F*	*Significance*
Complete model	1883.6	-	-	-	-
Cortical biomarkers only	2963.5	-5	-1079.7	3.554	0.012*
Lesion biomarkers only	4754.9	-15	-2871.3	3.150	0.003**
			**VOC**		
	Residual Sum of Squares	*Degrees of Freedom*	*Mean Square*	*F*	*Significance*
Complete model	1348.5	-	-	-	-

Cortical biomarkers only	3318.4	-10	-1969.9	3.506	0.006**
Lesion biomarkers only	4263.5	-14	-2915	3.706	0.002**

AHA, Assisting Hand Assessment; BRIEF, Behaviour Rating Inventory of Executive Function; GM, grey matter; SDQ, Strengths and Difficulties Questionnaire; TVPS, Test of Visual Perception Skills; VOC, vocabulary; WM, white matter; WR, Word reasoning
